# Multilayer Arrays for Neurotechnology Applications (MANTA): Chronically Stable Thin‐Film Intracortical Implants

**DOI:** 10.1002/advs.202207576

**Published:** 2023-03-19

**Authors:** Christian Böhler, Maria Vomero, Marisol Soula, Mihály Vöröslakos, Maria Porto Cruz, Rickard Liljemalm, György Buzsaki, Thomas Stieglitz, Maria Asplund

**Affiliations:** ^1^ Department of Microsystems Engineering (IMTEK) University of Freiburg 79110 Freiburg Germany; ^2^ BrainLinks‐BrainTools Center University of Freiburg 79110 Freiburg Germany; ^3^ Neuroscience Institute, Langone Medical Center New York University New York 10016 USA; ^4^ Department of Physiology and Neuroscience, Langone Medical Center New York University New York 10016 USA; ^5^ Department of Neurology, Langone Medical Center New York University New York 10016 USA; ^6^ Bernstein Center Freiburg University of Freiburg 79110 Freiburg Germany; ^7^ Department of Microtechnology and Nanoscience Chalmers University of Technology Gothenburg SE‐41296 Sweden; ^8^ Division of Nursing and Medical Technology Luleå University of Technology Luleå 97187 Sweden; ^9^ Freiburg Institute for Advanced Studies (FRIAS) University of Freiburg 79110 Freiburg Germany

**Keywords:** bioelectronics, conducting polymers, chronic recordings, flexible probes, neurotechnology, tissue‐device interfaces

## Abstract

Flexible implantable neurointerfaces show great promise in addressing one of the major challenges of implantable neurotechnology, namely the loss of signal connected to unfavorable probe tissue interaction. The authors here show how multilayer polyimide probes allow high‐density intracortical recordings to be combined with a reliable long‐term stable tissue interface, thereby progressing toward chronic stability of implantable neurotechnology. The probes could record 10–60 single units over 5 months with a consistent peak‐to‐peak voltage at dimensions that ensure robust handling and insulation longevity. Probes that remain in intimate contact with the signaling tissue over months to years are a game changer for neuroscience and, importantly, open up for broader clinical translation of systems relying on neurotechnology to interface the human brain.

## Graphical abstract

Multilayer flexible probes based on polyimide allow high‐density intracortical recordings to be combined with a long‐term stable tissue interface. Probes can reliably record single units over 5 months with a consistent peak‐to‐peak voltage. Their robustness in handling and insulation longevity open up for broader clinical translation of systems relying on neurotechnology to interface the brain.

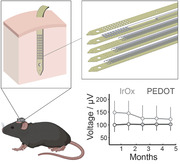

## 1 Introduction

Increasing the level of detail in neural recordings may advance the frontiers for our understanding of the brain in health as well as in disease. Microsized electrodes in intimate contact with neural tissue can bi‐directionally interact with neurons in the brain, making them indispensable as tools for studying the nervous system^[^ ^1^, ^2^, ^3^, ^4^ ^]^ and furthermore as components in future bioelectronic applications.^[^ ^5^, ^6^ ^]^ Rapid development on neural signal processing makes it clear that the hardware, particularly the implantable part of the interface, is a main bottleneck for resolution.^[^ ^4^, ^7^ ^]^ Even when the signals can be enhanced, filtered, and further decoded, improved quality of the raw data is decisive for progress in information extraction. Improved quality, in short, means sampling more neurons, with greater spatial resolution and over longer periods of time. Probes that remain in intimate contact with the signaling tissue over many months, possibly even a complete life cycle in small animals, certainly would be a game changer.^[^ ^8^ ^]^

Neurotechnology today massively under‐sample the brain. 1 mm^3^ of cortex contains ≈50 000 neurons, while a typical microfabricated probe provides only 16 sites to cover this entire recording space.^[^ ^4^, ^9^ ^]^ Thus, more electrodes are needed but importantly, also an increase in electrode packing density. Active probes are one solution, whereby electrode numbers can be increased from a few tens to several hundred.^[^ ^10^, ^11^, ^12^ ^]^ A remaining hurdle is nevertheless to maintain high quality recordings over time. In reality, glial scarring, inflammation, and neuronal loss can be expected to accompany all implantable neurotechnology within months.^[^ ^7^, ^13^, ^14^, ^15^, ^16^, ^17^ ^]^ Substantial effort has been invested into improving the biocompatibility of neural probes in order to preserve functional neuronal circuits in their vicinity. Particularly efficient in this respect appears to be the combination of the attributes “flexible” and “small cross‐sectional area.”^[^ ^8^, ^18^, ^19^, ^20^, ^21^ ^]^ For increasingly soft and flexible materials (e.g., SU‐8, polyimide (PI) or Parylene), exchanging silicon as a substrate, long‐term stable sampling of neural signals has been demonstrated over many months, e.g., by Chung et al. (4 months),^[^ ^18^ ^]^ Luan et al. (5 1/2 months),^[^ ^19^ ^]^ Zhao et al. (300 days)^[^ ^21^ ^]^ and even up to a year reported by Zhao et al. (2021).^[^ ^8^ ^]^ A remaining question is how to combine flexibility and small dimensions with the need for higher electrode integration density?

We here demonstrate how the spatial resolution of intracortical flexible shanks can be increased by adapting well‐established PI microfabrication processes to a multilayer assembly based on early own approaches.^[^ ^22^, ^23^ ^]^ The MultilAyer NeuroTechnology Array (MANTArray) is equipped with high‐performance electrode materials poly 3,4‐ethylene‐dioxythiophene/polystyrene sulfonate (PEDOT/PSS) and sputtered Iridium Oxide (SIROF). These support sensitive recordings and also perform robustly under stimulation. We show that the electrodes are capable of injecting 12 µA (or 54 nA µm^−2^) over at least one billion pulses, but their actual capability reaches up to 36 µA (200 µs, yielding a charge injection capacity of 3.2 mC cm^−2^).^[^ ^24^ ^]^ The resulting MANTArrays have 32 electrodes, of which 30 are densely packed at their front‐end (3×10 matrix, 24 µm pitch), providing exceptional spatial resolution. By routing connections in separate layer stacks, all tracks are integrated within the shank, thereby greatly reducing the outer dimensions down to a cross section of 10 × 78 µm^2^, which is even smaller than the typical active probe.^[^ ^25^ ^]^ The resulting shanks are biostable and integrate well with the surrounding tissue, as is here demonstrated by recording sessions lasting over 5 months. Our process thereby addresses a commonly reported shortcoming of flexible probes, namely the delamination of stacked/adjacent layers. The probes do not only endure months of implantation, but even survive subsequent explantation and removal of adherent tissue. In fact, the majority (93%) of the electrodes remained functional and for 62% the remeasured impedance was even close to the original curve. In summary, a well‐known process adapted to multilayer assembly solves the challenge of combining high‐density recordings (electrode pitch of 24 µm), with a reliable long‐term stable interface to the tissue.

## 2 Results

### 2.1 Microfabrication of High‐Density Flexible Probes

**Figure 1:**
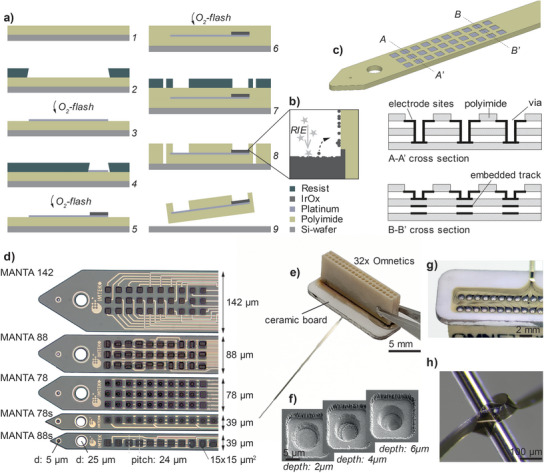
Microfabrication of high‐density flexible probes: a) Fabrication of flexible PI implants, comprising the imidization of a PI layer on a wafer (1), followed by lithographically patterning a photoresist (2) for subsequent metal deposition (3), which is here directly performed after activation of the surface with an O_2_‐plasma. A second photoresist mask (4) is used to define the SIROF electrode sites (5). All metal layers are insulated with a second PI‐layer (6), which is structured in a dry etching process via a photoresist mask (7) to define both the probe outline as well as the electrode‐ and interconnection openings (8). The probe is released from the substrate (9). b) Illustration of partial metal redeposition on the side‐walls of the PI during the final reactive ion etching (RIE) step while defining the electrode openings. c) Cross‐sectional view of the MANTArray technology, showing the stack of individual metal and PI layers at different positions along the probe. Layers and openings are not drawn to scale. d) Micrograph with the various design possibilities for MANTA multilayer probes. All probes have 5 insulating PI layers and 4 metallization layers but differ in the way that tracks are routed as follows: MANTA142—no tracks overlapping along the probe shaft; MANTA88—some overlapping of tracks; MANTA78: track overlapping with other tracks as well as electrodes; MANTA78s—single row variation of MANTA78, MANTA88s—single row variation of MANTA88. e) Fully assembled MANTA‐probe on the ceramic interface with an OMNETICS connector. f) SEM images of the MANTA78 design showing vias of different depth interconnecting the electrode sites to the feedlines in deeper metallization layers. g) Stable interconnection is achieved by directly soldering the probe to the ceramic board. h) The resulting probe is robust and yet very flexible. For instance, it is possible to tie a knot of the flexible shank without causing any damage to the probe.

As starting point, we used a well‐established process for the microfabrication of flexible PI based biosensors. In short, PI precursor is spin‐coated onto a carrier wafer and thereafter fully cured at high temperature under a nitrogen atmosphere (450 °C). Metal is either sputtered or evaporated and patterned using lift‐off photolithography. The final outline of the probes, as well as trenches for electrodes and contact pads, are defined by photolithography followed by dry etching. Similar processes (**Figure** **1**a) have previously been used for micromanufacturing a large variety of neurotechnological devices, e.g., intraneural, epicortical, spinal cord, and intracortical interfaces, as well as retinal implants.^[^ ^20^, ^26^, ^27^, ^28^, ^29^, ^30^, ^31^, ^32^, ^33^, ^34^, ^35^ ^]^

With the goal of making probes whose surfaces could be densely covered with electrodes, several adaptions were made to the base recipe (Figure 1b,c). First, we extended the process from the typical two‐layer design to five individual insulation layers (each 2 µm) and four separate metallization layers (each 100 nm). This way, it was possible to rout all connection lines on a 10 µm stack without adding to the width of the finished shank. Second, we substantially reduced the distance between connection lines. We concluded that 3 µm wide lines at a 6 µm pitch was the limit for reliable lift‐off clearance. It should here be noted that conductivity of the thin lines (3.6 kΩ cm^−1^ for 100 nm thick, 3 µm wide tracks) is not the limiting factor, but it is the edge clearance of the lift‐off process that determines the smallest possible dimensions. Challenges for the multilayer process are to maintain precise alignment throughout many lithography steps, ensuring that dry‐etched contours remain vertical, generating bonds connecting all layers, as well as defining and metallizing the smallest/deepest structures.

To analyze how reduced safety margins for defects and misalignment influence yield and reliability, we implemented three design variations (Figure 1d), using the same line‐to‐line clearance and layer thickness, but varying the way electrodes and connection lines were distributed (see also Figure S1, Supporting Information). Manta142 has the largest safety margins but only 20% of the tip is effectively used for electrodes, compared to 40% for the most compact design – Manta78. The latter has the smallest tolerances, tightest packing of lines and furthermore the smallest via footprint – a cylinder of 6 µm diameter, 2–6 µm deep. Manta88 represents a middle‐way, where directly overlapping tracks are separated by at least 2 layers (4 µm) of PI, to reduce potential crosstalk, and a larger via (6×15 µm^2^ wide, 2 µm deep) to increase process yield. The layouts furthermore differ in the placement of electrodes: for Manta88 and Manta142 they are at different depth, while for Manta78 all electrodes are part of the most superficial metallization layer. Finished probes were interconnected with an OMNECTIS and by a custom‐made ceramic board featuring through holes (Figure 1e,g).

Successful alignment and etching were the results of multiple process iterations optimizing parameters for the specific RIE machine and resist. Our patterning process resulted in etching and metallization (static evaporation) even of the narrowest vias with the largest aspect ratio (∅ 6 µm, depth 6 µm), as shown in Figure 1f. Further reduction of the via footprint would not have been feasible with the current processes as this is limited both by the ability of the metallization to reach into the deeper trench, and the challenges associated with etching straight walls deep into the PI (some overcut is inevitable). PI to PI bonding was addressed by high power oxygen plasma activation (O_2_‐flash at 100 W) immediately before each deposition step, to generate a strong bond to the subsequent layer. As shown in Figure 1h, the resulting probes are highly flexible and can, e.g., be tied into a knot, without damaging/creasing the device.

### 2.2 Electrode Materials for Recording and Stimulation

**Figure 2:**
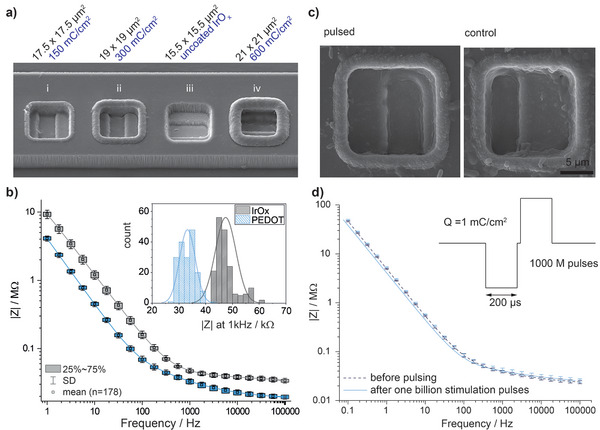
Electrode materials for recording and stimulation: a) SEM showing MANTA probes with different options for electrode materials. The electrode base consists of SIROF which either can be used as is iii), or alternatively be functionalized with PEDOT/PSS in an electrodeposition process. The thickness of the PEDOT‐layer is precisely controlled by the deposition charge applied, here shown for i) 150 mC cm^−2^, ii) 300 mC cm^−2^, and iv) 600 mC cm^−2^. PEDOT/PSS covers the underlying SIROF but furthermore climbs the walls of the electrode trench, even so the walls of the trench predominantly are insulating. Thus, it is important to precisely control the growth to avoid shorting of electrodes over the PEDOT‐layer, as would be the case for deposition charges exceeding 600 mC cm^−2^. b) Exemplary impedance measurements showing impedance from all electrodes and two groups of probes, featuring SIROF or PEDOT/PSS. Standard deviations are calculated across 178 electrode sites across multiple probes, and the histograms in the inset furthermore show the impedance distribution at the 1 kHz point. It is clear that all electrodes with the same material have close to identical impedance. Furthermore, PEDOT/PSS results in the lowest impedance, which likely is the combined effect of the large capacitance and the geometrical enlargement added by the edge growth. c) SEM images of a PEDOT coated electrode site after applying 1 billion stimulation pulses (1 mC cm^−2^) in comparison to a nonpulsed control. d) Comparison of the impedance spectrum before/after applying 1 billion stimulation pulses shows that characteristics barely changed (mean with standard‐deviation as error bars, *n* = 10).

The electrode pads (platinum 15×15 µm^2^) were coated with additional material (SIROF and PEDOT/PSS) to enhance their recording and stimulation properties (**Figure** **2**a,b). PEDOT/PSS was added by electrodeposition, either in parallel by shorting all electrodes on a probe (Figure S2, Supporting Information), or by sequential deposition (one electrode at the time). The latter was initially performed to confirm that each electrode was truly individually connected without short‐circuits, pinholes, or detachment between layers. Any of these imperfections would immediately become apparent as coatings would not be limited to the connected electrodes, coverage would be inhomogeneous, or growth would occur as a rim under the top insulation. None of these typical failures could be observed which supports the integrity of the insulating barriers, as well as the efficiency of the method used for metallizing the vias. In fact, the use of ultrathin layers (2 µm) and multilayer stacked designs appears unproblematic in this respect.

Films formed under identical conditions were highly reproducible, with higher total deposition charge resulting in thicker films and lower impedances (Figure S3, Supporting Information). The lower impedances of thicker layers are coupled to the growth pattern of PEDOT/PSS, which covers the SIROF as well as the edges of the electrode trench, resulting in a cup‐shaped electrode with an increasing rim overgrowth at increased deposition charge (Figure 2a). This is likely related to redeposition of metal residues that occurs in the final RIE step (Figure 1b), where the most superficial SIROF layer will be exposed to the reactive plasma as the electrode is etched open. Consequently, a thin layer of Ir may be removed and redeposit within the narrow trench, providing a fine metal coverage of the walls. On its own, this layer would have poor conductivity but, merged with PEDOT/PSS, it forms a cohesive and conducting wall. The wall‐growth creates a conducting path to the surface of the probe and consequently an enlarged electrode area in comparison to the electrode footprint on the surface. Further over‐growth can be avoided by controlling the deposition charge density (Figure S3, Supporting Information). In all further depositions, we grew films to a total of 225 mC cm^−2^, which provided a reasonable balance between impedance reduction and clearance to adjacent sites. Electrochemical impedance spectroscopy (EIS) and cyclic voltammetry (CV) confirmed that the yield of functional electrodes per probe was overall high (more than 93%, *n* = 416), and furthermore that electrodes of the same material have highly reproducible impedance spectra as shown for PEDOT/PSS and SIROF electrodes in Figure 2b. The 1 kHz impedance of electrodes (inset Figure 2b) was on average 33.18 (±2.95) kΩ for PEDOT/PSS and 47.46 (±3.87) kΩ for SIROF, with the cut‐off frequency at 130.36 (±10.65) and 347.97 (±58.26) Hz, respectively (mean over *n* = 178 with standard deviation). Delamination under stimulation is reportedly one of the major challenges for thin‐film devices, as any irreversible electrochemical reaction will rapidly impact the entire electrode bulk.^[^ ^27^ ^]^ SIROF stabilized PEDOT/PSS reduces the already low impedance provided by the SIROF layer and allowed us to reach a maximum safe charge injection of 3.2 mC cm^−2^ (36 µA, 200 µs).^[^ ^24^, ^36^ ^]^

The long‐term stimulation performance was evaluated in vitro, with a pulsed stress test, set so that the injected charge per pulse would be one third of that necessary to reach water electrolysis. This level of charge injection (12 µA, 1 mC cm^−2^) was maintained over 1 billion pulses in saline (bi‐phasic charge balanced, 800 Hz repetition frequency). By inspecting electrodes with high resolution scanning electron microscopy (SEM) and EIS before and after the stress test, we confirmed that the electrodes endured stimulation well, as no significant changes were apparent, neither in the high‐resolution images, nor in their electrochemical properties (Figure 2c,d). All stimulated films remained adherent, and no signs of delamination or other damage were evident. In fact, EIS taken before and after stimulation (Figure 2d) showed nearly perfect superimposition. Polarization was monitored during the pulse test, with no significant changes over the 1 billion pulses tested (Figure S4, Supporting Information). We can thus confirm that SIROF‐stabilized PEDOT/PSS acts as an extremely stable complex.^[^ ^36^, ^37^ ^]^

### 2.3 High‐Density Layout is Not Compromised by Cross‐Talk

**Figure 3:**
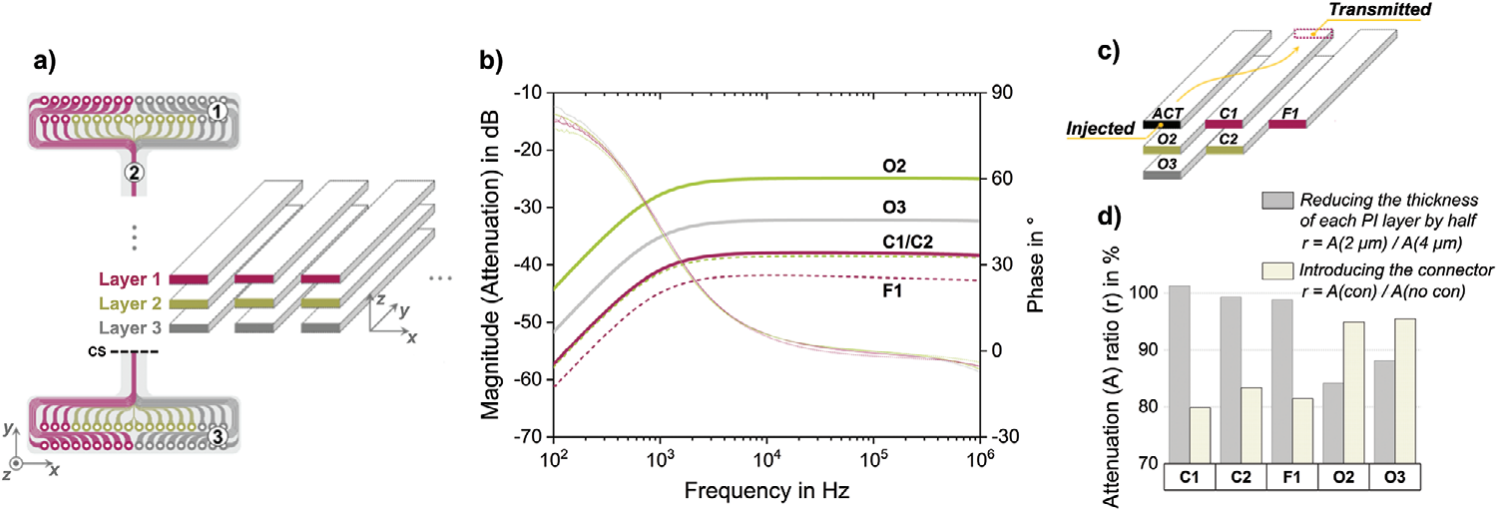
High‐density layout is not compromised by cross‐talk: a) Schematic of the PI‐based test structure used to quantify crosstalk in multilayered structures. Labels 1 and 3 refer to the pad areas and label 2 refers to the 4 cm long structure of parallel tracks running along three routing layers. CS stands for cross‐sectional view. Not to scale. b) Bode plot of signal attenuation for different relative positions between active and passive tracks, according to the schematic in (c). For these measurements, each PI layer had a thickness of 2 µm and no connector was considered. c) Schematic depicting the crosstalk measurement principle. Attenuation is given as a ratio between injected and transmitted signals. ACT stands for the active line. C_1_ and F1 stand for close and far tracks in layer 1. O_2_ and C_2_ stand for overlapping and close tracks in layer 2. O_3_ stands for overlapping track in layer 3. Not to scale. d) Effect of reducing PI layer thickness from 4 to 2 µm and effect of introducing the OMNETICS connector on the measured crosstalk. The effect is quantified as the attenuation ratio at 1 kHz for 2 versus 4 µm thick PI layers and for presence versus absence of connector (ratio calculation specified in the legend).

An important point to consider when designing high‐density probes is the implication smaller safety margins will have on signal quality, particularly in terms of crosstalk between adjacent routing lines. Crosstalk is here defined as the undesired electrical coupling between an active line (carrier of the true signal) and an adjacent passive line (carrier of the coupled signal),^[^ ^38^ ^]^ and it is well distinguished from the volume conduction at the tissue level.^[^ ^39^ ^]^ A multilayered PI‐based test structure composed of parallel tracks running along three routing layers was specifically designed to assess crosstalk as a function of the relative position between tracks (**Figure** **3**a,b). Each PI layer had a thickness of 2 µm (same thickness used for the MANTAs), and the tracks within each layer were all spaced by 10 µm (Figure 3a). The crosstalk measurements were performed over the relevant frequency range for spike activity (100 Hz to 1 MHz) while immersing the test structures in phosphate buffered saline (PBS) to mimic the in vivo environment.^[^ ^24^ ^]^ This setup represents the worst‐case scenario, as the passive line was left floating, simulating a scenario where the electrode interfaced by the passive line would be completely decoupled from the signal source due to breakage or glial encapsulation (infinitely high impedance).

Crosstalk was measured in terms of signal attenuation, where 100% signal attenuation between active and passive line corresponds to zero crosstalk (−∞ dB). The Bode plot showed a typical high‐pass filter profile with a decay of 20 dB per decade and corner frequency around 1 kHz (Figure 3b,c). The coupling was the strongest between directly overlapping tracks (O_2_) and significantly stronger between overlapping tracks within nonconsecutive layers (O_3_) than between adjacent tracks on the same layer (C1). Nonetheless, considering the worst‐case scenario, no more than 5% of the signal would be coupled into the passive line (−25 dB plateau for O_2_).

The use of an OMNETICS nanoconnector as an interface between the thin‐film probe and the recording system (as shown in Figure 1e,g) was also investigated with regard to crosstalk. Although it did not significantly aggravate the worst‐case overlapping scenario (O_2_), the connector did contribute to a boost in electrical coupling between adjacent lines (Figure 3d), as attenuation dropped to ≈80% for C1 and F1. Regardless, the coupling seen between perfectly overlaying lines belonging to consecutive layers is clearly the most substantial contributor to crosstalk. Even then, the signal coupling can be expected to be way below the worst‐case scenario of 5% when electrodes are properly in contact with tissue.

### 2.4 Longevity of Recordings with Flexible Probes

**Figure 4:**
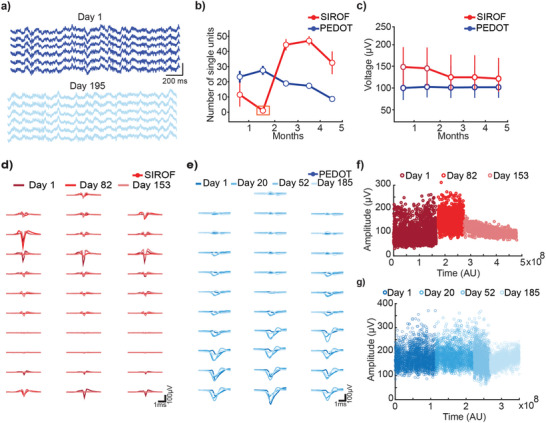
Longevity of recordings with flexible probes. a) Example LFP traces from channels 1:6 for day 1 and day 195 (PEDOT device). b) Average number of single units per month for PEDOT and SIROF electrodes over a 5 month recording period (at least 5 recording sessions/month). Orange box represents a period where little to no units were recorded from. c) Average single unit peak‐to‐peak voltage across months for PEDOT and SIROF electrodes (mean±SD is shown, at least 5 recording sessions/month, *n* = 15 and 34 sessions for SIROF and PEDOT electrodes, respectively, *p* > 0.05, Wilcoxon Rank Sum test). Average waveform of a neuron per channel on the d) SIROF flexible electrode and e) PEDOT flexible electrode. Darker colours are earlier days, while the lighter colours correspond to last days of recording. f,g) Peak‐to‐peak amplitude of spikes from a neuron over time (each dot represents a spike amplitude from a neuron) in the f) SIROF flexible electrode and g) PEDOT flexible electrode.

In vivo implantation of flexible probes featuring both PEDOT and SIROF electrodes in the parietal cortex of wild‐type mice confirmed the stability of the probes seen in vitro. The local field potential and single unit activity were recorded from the 32 channels over more than 150 days in freely moving mice (**Figure** **4**a). These recordings were stable and were not disrupted by noise. Baseline RMS of our measured noise was 15.51 ± 3.1 µV on day 1 and 9.38 ± 1.2 µV on day 195 (mean±SD, PEDOT probe). Both electrode materials could record 10–60 single units for ≈5 months (Figure 4b). The SIROF electrode was unstable during the first month (orange box Figure 4b) recording 1–10 single units. However, it stabilized after 7 weeks and was able to record from 30+ neurons for 4 months thereafter. This may highlight the importance of waiting 6 weeks prior to recording to allow the electrode to stabilize and the tissue to heal.^[^ ^40^ ^]^ The average peak‐to‐peak voltage of all recorded single units did not change over time (*n* = 15 and 34 sessions for SIROF and PEDOT electrodes, respectively, *p* > 0.05, Wilcoxon Rank Sum test). Importantly, both devices were able to record neurons for 185 (PEDOT, Figure 4e,g) and 153 days (SIROF, Figure 4d,f). The amplitude of these recorded units were consistent over months (Figure 4c) and their waveforms have similar amplitude, duration, and spatial location across the recording period (Figure 4d,e). This suggests the probe micromovements are minor and it is possible to record several neurons for over 5 months, presenting a clear advantage over silicon‐based probes for chronic recordings. To further assess the quality of the recorded neurons, we looked at various features of putative single units over the 5‐month recording periods. Firing rate, firing rate instability, spike count and refractory period violation did not change significantly over 5 months (*n* = 15 and 34 sessions for SIROF and PEDOT electrodes, respectively, *p* > 0.05, Wilcoxon Rank Sum Test) in PEDOT and SIROF electrodes (Figure S5a–d, Supporting Information). This verifies that viable neurons remained in the area surrounding the probe, speaking for a stable tissue integration as expected based on the previously documented biostability and structural biocompatibility of similar probes.^[^ ^20^ ^]^

**Figure 5:**
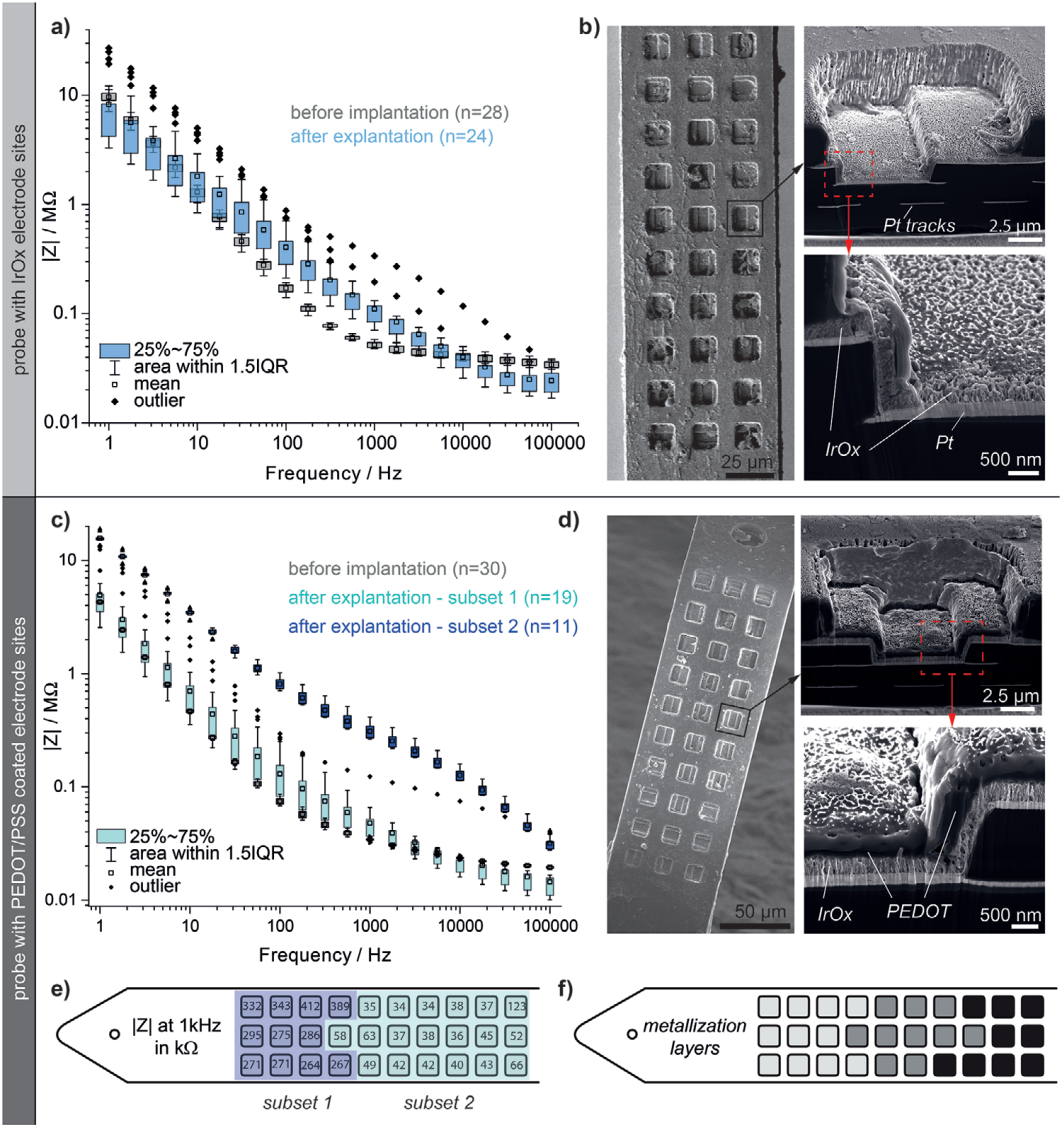
Longevity of flexible probes during implantation and explantation: a) EIS of a SIROF probe measured before implantation (*n* = 28) and after explantation (180 days in vivo, *n* = 24). b) SEM image of the same explant showing an overall intact probe, partially covered in biological residues. Cross‐sectional images show well embedded Pt‐tracks inside the PI, the IrOx layer can clearly be identified on top of the Pt‐substrate without any signs of delamination or corrosion. c) EIS comparison for an explanted probe coated with PEDOT/PSS (*n* = 30). d) SEM images of the explanted PEDOT probe reveal adherent PEDOT coatings on the SIROF substrate, both on the surface as well as inside the via connecting two metallization planes. e) The two distinct impedance groups measured for the explant in (c) can be mapped to specific locations on the probe. f) Comparison with the metallization layers of the probe shows a correlation between higher impedance (i.e., subset 2) and the deepest metallization layer (light gray) located at the tip of the probe. Boxplots show the interquartile range (IQR) as box, whiskers denote the area within 1.5 IQR, the central mark represents the mean and outliers are marked with diamonds.

After over 180 days of recording, we explanted probes for subsequent electrochemical characterization and SEM analysis. Careful cleaning of the skull surface and head stage with saline and gentle aspiration of debris was performed prior to removing the probe. The probe was slowly removed with the same device used for insertion. The explants were first stored in saline for shipping, thereafter, immersed in a protein removing agent over 7 days in order to reduce the biological residues before CV and EIS. **Figure** **5**a shows a comparison for the impedance data of the SIROF probe measured prior to implantation, as well as after explantation. While four channels featured high impedance values (> 2 MΩ at 1 kHz, not shown in Figure 5) due to damaged solder interconnections, all remaining sites were found to exhibit rather similar impedance values (|Z|_1 kHz_: 111 ± 56 kΩ) in comparison to the pristine state (|Z|_1 kHz_: 52 ± 3 kΩ). This is well in line with our observations from the recording data, illustrating that the probe remained intact over the 180 days in vivo. SEM revealed no signs of delamination nor degradation of the electrode materials (Figure 5b). Cross‐sectional imaging using a focused ion beam (FIB‐SEM) moreover showed well adherent layers of Pt and PI throughout the entire probe cross‐section.

The impedance data measured from the explanted PEDOT probe showed two distinct characteristics (subset 1 and subset 2 in Figure 5c). The first group (*n* = 19 electrodes) was found to exhibit similar impedance values as compared to the pristine state (|Z|_1 kHz_ = 48 ± 21 kΩ). The second subset (*n* = 11 electrodes, which correlated with electrodes connected over the deepest metallization layer, Figure 5e,f) exhibited a six times higher impedance (|Z|_1 kHz_ = 309 ± 53 kΩ). These electrodes were still functional although with reduced quality. Under SEM the probe appeared intact, there were, e.g., no signs of delamination between the metal and PI. The FIB‐SEM images in Figure 5d show well‐embedded tracks under the electrodes. Adherent PEDOT coatings could be identified on all individual electrode sites, including on the sidewalls and inside the vias (Figure 5d), with the only visible change being a few cracks in the PEDOT films, as is expected after repeated dehydration/wetting and SEM under high vacuum (Figures S6 and S7, Supporting Information).

## 3 Discussion

Recent studies on intracortical implants highlight increased electrode density, small‐cross section and device flexibility as central aspects for neurotechnological progress.^[^ ^10^, ^11^, ^12^, ^18^, ^19^, ^20^ ^]^ Here we show i) how these attributes can be combined with high electrode density and ii) how this supports maintained signal quality over long implantation times (i.e., 5 months or more). As an alternative to exploring completely novel fabrication methods, we chose to refine an established photolithography process for PI microfabrication on wafer level, thereby leveraging over 30 years of experience addressing interlayer adhesion, interconnection, and functionalization of such probes.^[^ ^20^, ^26^, ^27^, ^28^, ^29^, ^30^, ^31^, ^32^, ^33^, ^34^, ^35^ ^]^ The process can be adapted to large‐scale manufacturing and industrial batch production including parallel coating of PEDOT/PSS to further enhance the electrode properties.

The MANTA devices presented are passive PI‐based shanks designed for the purpose of creating a stable tissue‐electrode interface and to reliably record neural activity over several months. Quality of recordings relies on electrodes located as close as possible to the target neurons, ideally as silent and undetectable observers, which necessitates that the tissue reaction is kept at a minimum. Our goal was to accomplish exactly this, without sacrificing functionality, i.e., recording resolution.

The multilayer approach allowed us to increase the electrode density at their tip (600 µm^2^ per channel), even with respect to typical active (i.e., silicon‐based) probes,^[^ ^25^ ^]^ while remaining highly flexible and more structurally biocompatible than their stiff counterparts. This presents the highest electrode density achieved for flexible probes fabricated with conventional photolithographic processes as summarized in **Table** **1**.

**Table 1:** Comparison of performance metrics for high‐density flexible neural probes

Material	Wang 2020^[^ ^41^ ^]^	Pimenta 2021^[^ ^42^ ^]^	Joo 2019^[^ ^43^ ^]^	Zhao 2022^[^ ^21^ ^]^	Liu 2021^[^ ^44^ ^]^	Srikantharajah 2021^[^ ^45^ ^]^	Leccardi 2019^[^ ^46^ ^]^	MANTA 39	MANTA 78	MANTA 88
	PaC	PI	PI	SU‐8^a)^	PaC	PaC^b)^	PaC	PI	PI	PI
Thickness [µm]	20	24	14	1	9	10	21	10	10	10
Total # of electrodes	64	32	32	128	16	16 (32)	8	12	32	32
# of shanks	8	1	2	8	1	4	1	1	1	1
# of electrodes per shank	8	32	16	16	16	4 (8)	8	12	32	32
|Z| at 1 kHz [kΩ]	691	241		100	450	26	194	33	33	33
|Z| at 1 kHz in [MΩ µm^2]^	488.2	18.9		62.5^(3)^	317.9	**2.9^(1)^**	1522.9	7.4^(2)^	7.4^(2)^	7.4^(2)^
Electrode dimension [µm]	Ø 30	Ø 10	Ø 20	25×25	Ø 30	Ø 12	Ø 100	15×15	15×15	15×15
Pitch [µm]	70	**15^(1)^**	40^(3)^	60	60	200 (50)	200	24^(2)^	24^(2)^	24^(2)^
Total surface per shank [1000 × µm^2]^	62.4	14.6	25.6	36.2	76.0	61.2 (26.6)	500	10.9	18.0	21.6
Surface covered by electrodes [µm^2^]	5656	2513	5027	10 000	5656	452 (904)	62 800	2700	7200	7200
Surface coverage by electrodes [%]	9	17	20	28^(3)^	7	1 (3)	13	25	**38^(1)^**	33^(2)^
Surface per electrode [1000 × µm^2^]	7.80	**0.46^(1)^**	1.60	2.26	4.75	15.3 (3.32)	62.5	0.91	0.60^(2)^	0.68^(3)^
Volume per shank [1000 × µm^3^]	1250	351	358	36	684	612 (279)	10 510	109	192	216
Volume per electrode [1000 ×µm^3^]	156	11	22.4	**2.26^(1)^**	42.8	15.3 (34.8)	1314	9.07	6.00^(2)^	6.76^(3)^
Cable cross‐section [µm^2^]	3000	3600	1120	60	720	1000 (945)	9450	390	780	880
Cross‐section per electrode [µm^2^]	375	113	70	**3.8^(1)^**	45	250 (118)	1181	32.5	24.4^(2)^	27.5^(3)^
Validation in vivo [days]	Over 35	Acute	On day 95	Over 145/290	Acute	Acute	Acute	—	—	Over 195

^a)^ Data for probe type I (geometry which has been validated in vivo);

^b)^ Values in parentheses describe a second generation, which has been mentioned but not validated in the paper;

^(1–3)^ indicate top three ranking with respect to performance.

In previous work,^[^ ^20^ ^]^ we demonstrated that flexible neural implants do not necessarily need to reach subcellular dimensions to be fully integrated within the host tissue and function for long periods of time, as evidenced by histology in combination with spike recordings. In fact, probes with a cross section of 12 × 100 µm^2^ (aka PIXI‐100) were still able to record single units for at least 3 months after implantation. In view of this excellent tissue integration and targeting stable electrophysiological performance over even longer periods, the MANTAs were designed to be slightly smaller than the PIXI‐100s, and still have almost three times the number of electrodes (32 vs 12). This downscaling represents a giant leap forward. To provide a figure of merit, **Table** **2** summarizes how track pitch (20 vs 6 µm) and number of separate routing layers (1–3) impact shank width, here using numbers that reflect typical PI processing scale, also our own previous work.^[^ ^20^, ^26^, ^35^ ^]^ The difference from the most simplistic process to the MANTAs, is at least a three times reduced shank width. “At least” is here appropriate as Table 2 neglects that tracks and electrodes often are routed in one and the same layer, meaning substantially widening the shanks even further emphasizing the value of layering.

**Table 2:** How scaling of tracks and multiple layers influence shank width. It is here assumed that electrodes are in a separate layer connected with vias and neglecting any added contribution of the via to the width. Commonly, electrodes and tracks share a layer in which case shanks would be much wider. In either case, numbers here are an underestimation

Layers	Track to track spacing 10 + 10 µm	Track to track spacing 3 + 3 µm
1	>650	>195
2	>330	>100
3	>230	>67

The rationale for making probes with high electrode integration density is to enable recordings simultaneously from as many electrodes as possible closely spaced on the surface of the probe, for instance to use spatial over‐sampling to facilitate spike‐sorting.^[^ ^42^ ^]^ Compactness is in addition desirable to minimize the volume of foreign material that needs to enter the brain. Another way to see it is that every added electrode comes at a “cost” in terms of increased implant volume and cross‐section of the cable. A microfabrication process for high‐integration density allows us to minimize this “cost.” The smallest possible feature size is determined by the efficiency of the electrode material and the precision of the microfabrication process, whereby electrochemical and geometrical comparisons are important for evaluating if a process truly has increased the possible integration density.

Our study focused on achieving as high integration density as possible on a penetrating probe shank using standard photolithographic patterning of multilayer thin‐film polyimide. Other groups have targeted high integration density using alternative flexible substrate materials (Parylene, SU‐8) or patterning technology (e‐beam lithography). Most similar to ours is the study by Pimenta et al. where 32 electrodes are embedded on one shank in a multirow configuration.^[^ ^42^ ^]^ The team of Wang et al.^[^ ^41^ ^]^ reported on an 8‐shank Parylene device with a total of 64 electrodes, and the team of L. Frank presents two studies with polyimide based multishank high‐density devices,^[^ ^18^, ^43^ ^]^ with typically 16 electrodes per shank. The team of C. Xie reported on SU‐8 based NET‐electrodes^[^ ^19^, ^21^ ^]^ with up to 8 shanks per module, and with 16 or 32 electrodes per shank in single‐/dual‐row configuration, respectively.

In order to provide a transparent comparison to these other processes and studies, we defined a set of key measures, detailed under the Supporting Information: “Determination of performance metrics for high‐density neural probes.” For comparing across studies, we choose to compare at single shank level, as our goal was to increase electrode numbers by increasing integration density, and not scaling of technologies to include more but distributed electrodes. From Table 1, it is clear that the smallest volumetric “cost” per electrode is represented by the SU‐8 probes which are fabricated using e‐beam lithography and using an exceptionally thin substrate of only 1 µm.^[^ ^19^, ^21^ ^]^ Even so the MANTAs are among the thinnest flexible probes, we choose not to reduce the layer thickness to this extent (which in our case would mean 200 nm per insulation layer). Nevertheless, we set out to show that similar high‐density is possible using conventional lithographic processes, benefitting from batch processing and the high biostability of high‐temperature cured polyimide. The MANTA versions present the overall highest percentage surface coverage with electrodes, and the second lowest volumetric cost, as compared to the e‐beam fabricated SU‐8 probes or other high‐density/multilayer probes (Table 1). Based on the introduced ranking in Table 1, highlighting the top three values (index 1–3) with respect to high‐density performance, it can moreover be seen that the MANTA probes feature an overall high‐performance score. From this we conclude that our probes represent the highest integration density for photolithographically fabricated probes and compare well to the NET‐probes in terms of the efficiency by which the recording area of the probe is covered with electrodes. If more electrodes per volume are needed, implantation of multiple shanks one after the other offers a more resilient approach than a multishank device, since single shanks can be aligned to the target structures specifically and, in case of failure, can be exchanged on an individual base. Thereby, functionality of remaining shanks can be maintained instead of sacrificing the whole implant. In addition, our electrodes show a very low 1 kHz impedance. Important to note is moreover that our study shows that the layer stack, enabling the high integration density, remained intact over at least 5 months of recordings, while many studies focus on acute tests. High‐density microfabrication in particular challenges longevity aspects and to settle this concern we aimed for a long implantation time.

Successful miniaturization is not only a function of patterning resolution and alignment, but furthermore precludes materials that remain effective at reduced scale. To demonstrate this our study includes a stimulation stress‐test and cross‐talk analysis, two factors of vital importance for reliability of probes. The electrode sites were coated with PEDOT/PSS (over SIROF) and stimulated with 1 billion biphasic pulses (1 mC cm^−2^) after which they showed no signs of delamination or failure. Thus, there is no need to preconfigure the electrodes for recording or stimulation as they are efficient in both modalities. Based on crosstalk characterization between adjacent and parallel tracks, at different distances, we could validate that crosstalk is not a major concern, and its potential contribution to signal loss is insignificant in the multilayer designs shown here. Although commercially available connectors still are a limiting factor for the further increase of the total number of electrodes, it is in the future possible to exchange them for CMOS‐based backends using processes described by others.^[^ ^18^, ^47^ ^]^

The main technical questions we aimed at addressing are

1) Which design considerations that are most critical for reaching high electrode density on flexible intracortical shanks?
2) If thin (cross section < 1000 µm^2^) PI‐based shanks are sufficiently robust to endure implantation over longer times?

With respect to the first question, we conclude that all our design variations produced well‐functioning probes, including those with the smallest vias. Further reduction of feature size and tolerances would likely negatively impact the yield of this specific manufacturing protocol. To enable even further reduction in size, the metallization of the smallest vias (Ø 6 µm, depth 6 µm) could be further optimized. For instance, the dry etch step could be tuned to provide a slanted, instead of vertical, profile of the via walls and electroplating could be added to further improve the sputtered metallization.

The second question is raised by the frequent observation that adhesion between PI layers is a weak spot of flexible microtechnology. In contrast to this, we find that microfabricated PI probes are robust given that certain critical steps in the process are met. PI to PI bonding was here accomplished by high power oxygen plasma activation immediately before each deposition step, to generate a strong bond to the subsequent layer. Furthermore, the materials stack at the electrodes is crucial for stability under stimulation, which was here solved by combining Pt, SIROF, and PEDOT/PSS. Not only did the MANTAs remain intact over 6 months of implantation, but they even endured the harsh treatment of explantation, storage with adherent tissue, shipping, and thereafter exposition to massive cleaning procedures to remove dried biological material adhering to the probes. Impedance measurements and high‐resolution FIB‐SEM analysis postexplanation clearly demonstrate that the electrodes were still in functional condition. There were no signs of delamination neither at the PI‐to‐PI interfaces, nor at the interfaces between electrodes and PI, demonstrating excellent long‐term stability also from the technical point of view in addition to the stable electrophysiological performance observed.

Naturally, clinical use of neurotechnological implants would require much longer time spans than shown here, ideally devices remaining functional for the rest of a patient's life. It is hard to predict via short‐term models how flexible implants would perform in a brain after a few years, and it is challenging (financially and logistically) to justify such a lengthy in vivo study in academic settings. We here show data that support that PI‐based devices certainly are capable to perform reliably even under extreme conditions, that they can be safely explanted and that they are highly beneficial for addressing gliosis, three key aspects for clinical translation of neurotechnology. While regulatory and ethical considerations raise the bar when designing for human use, it should be noted that the scaling factor from man to mouse is connected with challenges as well.^[^ ^48^ ^]^ The more voluminous proportions of the human brain might even reduce the foreign body response to individual probes, analogously to the biomechanical analysis of epicortical grids for different species shown by Vomero et al.^[^ ^33^ ^]^

Another important aspect we would like to address here concerns the ability of a flexible implant to record single unit activity and “track” the same neurons over time, which would enable the study of specific neuronal pathways without interfering with them. Previous reports claimed to record from the same neurons over extended time even with rigid electrodes.^[^ ^18^, ^49^ ^]^ Unfortunately, there are no absolute criteria which would safely identify cluster‐separating based units over time as “same.” Behavioral criteria, such as stimulus evoked responses or place field stability, are not reliable because of the demonstrated “representational drift.”^[^ ^18^, ^49^, ^50^ ^]^ Waveform stability is a stronger criterion since the relationship between the recording sites and the somadendritic axis may remain the same over time. In case of small drifts, the relative depth distribution of the unit amplitude may remain the same and, thus, could be adjusted for.^[^ ^49^ ^]^ Yet, the somadendritic backpropagation of the action potential may also change with time, adding uncertainty to the unit amplitude profile identification. Further, if the firing rate of the neuron changes over time (as it has been shown to be the case with representational drift), the waveform also changes since waveform is very sensitive to activity level.^[^ ^51^ ^]^ Single unit stability and the Mahalanobis distances between clusters is another option.^[^ ^52^ ^]^ However, because of the representation drift and associated firing rate changes, single‐unit centers may change. Overall, while our physiological recordings confirm stable chronic recording of neurons, we would like to caution that ascertaining unit identity remains a further challenge.

## 4 Conclusion

We show here an adapted process for the fabrication of high‐resolution flexible probes, that are robust and versatile as neurotechnological tools for neuroscience. They complement the neurotechnological toolbox and address two of the challenges of contemporary active probes, namely, tissue interface stability and bi‐directional spatial resolution.

## 5 Experimental Section

### Probe Fabrication

MANTA probes were fabricated by advancing standard PI fabrication protocols to a multilayer design, comprising ultimately 5 PI and 7 metallization layers in a probe of 10 µm thickness. First a 2 µm thin layer of PI (U‐Varnish S, UBE corporation) was spincoated onto a carrier wafer and cured at 450 °C under nitrogen atmosphere (YES‐450PB8‐4PB/E, Yield Engineering Systems Inc.). A liftoff‐resist (AZ5214E, MicroChemicals GmbH) was subsequently used to pattern the first Pt‐layer (100 nm, static evaporation, Univex 500, Leybold). Prior to the metal deposition, the PI‐surface was activated in an O_2_‐plasma (30 s at 100 W, STS‐RIE) to ensure stable adhesion between the layers. After another O_2_‐plasma treatment, this metal layer was insulated with a second PI layer (2 µm), which was then partially opened by reactive ion etching using an O_2_‐plasma (100 W, STS‐RIE) and a resist mask (AZ9260, MicroChemicals GmbH) to define the first set of vias. A second Pt‐layer was afterward deposited (following again an O_2_‐plasma treatment and using the same parameters as previously described) to define the tracks and electrodes in the second metallization plane. The electrode sites were additionally coated with a sputtered iridium oxide (SIROF) layer (800 nm, 100 W, 15 sccm O_2_, Univex 500, Leybold) using again a lift‐off resist to define the electrode geometry. The same process steps (O_2_‐plasma, Pt + SIROF‐deposition, and PI‐insulation) were repeated twice to build up a total stack of 5 PI layers with 4 Pt‐metallization planes and three SIROF layers. The topmost PI‐layer was finally patterned using RIE to realize simultaneously the electrode openings above the SIROF sites, to open the Pt‐pads for solder interconnection at the backend of the probe, as well as to define the overall outline of the probe. For further use and characterization, probes were individually peeled off from the carrier wafer.

### Connector Assembly

Five layers of HTCC (high temperature co‐fired ceramic) precursor tape (44 000, ESL ElectroScience, PA) were cut and pressed at 27 MPa, resulting in a single layer roughly 1 mm thick. The outlines and holes through which the connector pins were meant to be inserted were laser patterned on the tape (DPL Genesis Marker, cab Produkttechnik GmbH, Germany). After an organic burnout at 550 °C and sintering at 1500 °C, silver‐palladium paste was used to metalize the laser‐structured holes. The paste was pressed into the holes from the top side of the ceramic and vacuumed from the bottom side, resulting in metallization of the inner walls of the holes. The paste was fired at 850 °C, followed by a grinding step on each side of the ceramic to remove the paste excess. The through‐hole connector (A79022‐001, OMNETICS Connector Corporation, MN) was then inserted into the ceramic and soldered. The solder bumps were grinded until flat. For mechanical stability and insulation, epoxy (EPO‐TEK 353ND‐T, Epoxy Technology Inc., MA) was spread around the edge between connector and ceramic. Finally, to mount the thin‐film on the connector stage, the pads of the thin‐film were aligned to the flat contacts on the ceramic and subsequently soldered using low‐temperature soldering paste. The solder bumps were protected and insulated with fast‐curing two‐component epoxy (UHU Plus Schnellfest, UHU GmbH, Germany).

### Electrochemical Characterization

All probes were characterized in vitro following standard test protocols^[^ ^24^ ^]^ Prior to EIS measurements, an electrochemical cleaning step was implemented, meaning all sites were shorted and CV‐cycled in PBS (0.01 m) at a scan rate of 100 mV s^−1^ with vertex potentials of −0.6 and 0.9 V. EIS data were subsequently measured at the open cell potential and using sinusoidal excitation signals with an amplitude of 100 mV pp in the frequency spectrum from 0.1 Hz to 100 kHz. The cutoff‐frequency was determined at a phase shift of −45 °C in the Bode plot.

### PEDOT Deposition

PEDOT/PSS coatings were polymerized electrochemically on top of the SIROF sites to ensure maximum process control and thus provide high quality PEDOT films. Deposition was carried out in an electrolyte containing 0.01 m EDOT and 5 mg mL^−1^ NaPSS using a potentiostatic deposition scheme with charge cutoff control to yield reproducible films. A three‐electrode configuration was used where electrode sites were either individually connected to the working electrode or shorted in groups of 8–11 sites. A stainless‐steel mesh (area >100x working electrode) was used as counter electrode and an Ag/AgCl electrode served as reference electrode during the potentiostatic deposition at a potential of 0.9 V. Prior to the PEDOT deposition, all sites were electrochemically cleaned and characterized. Directly following the coating step, probes were intensively rinsed with DI‐H_2_O, CV‐cycled in PBS to remove remaining deposition‐electrolyte from the coating and finally characterized again in fresh PBS.

A subset of probes (*n* = 73 electrode sites) was additionally used to investigate the possibility of parallel PEDOT deposition across shorted electrode sites, which will be crucial for future batch processing. Individual electrode sites were initially characterized, then shorted in groups (either 8, 10, or 11 sites) for the electrodeposition of PEDOT and subsequently characterized to assess the individual characteristics. EIS data of electrode sites coated one at a time (serial) or in parallel, did not show any significant differences evidenced by their 1 kHz impedance and cutoff‐frequency of 29.64 ± 0.34, 30.28 ± 0.43 kΩ, 131 ± 11 and 130 ± 12 Hz for serial and parallel coated electrode sites, respectively. The full impedance spectrum is provided in the supporting information (Figure S2, Supporting Information).

### Long‐Term Pulsing

A subset of probes was exposed to biphasic current pulsing in order to evaluate the long‐term stability of the probe/electrode coating under high stimulation load. The maximal charge injection was therefore initially determined for biphasic rectangular stimulation pulses with a pulse duration of 200 µs per phase. These measurements were performed with a Plexon Stimulator in a two‐electrode configuration where the current amplitude was successively increased during stimulation until the voltage drop measured across the electrode was observed to reach the water limits, representing the potential at which electrolysis begins to occur at the electrode interface. Long‐term pulsing was subsequently performed in the same setup using a current amplitude that corresponds to one third of the max charge injection, here 12 µA. Pulsing at this current was maintained at a pulse repetition frequency of 800 Hz until a total number of 1 billion stimulation pulses were delivered to the electrode. The voltage drop across the electrode was measured regularly during the pulsing time and EIS characteristics were recorded after reaching the target count of 1 billion stimulation pulses for comparison to the initial data set.

### Surgical Procedure

All experiments were approved by the institutional Animal Care and Use Committee at New York University Langone Medical Center. C57BL/6 adult mice from JAX lab were kept in the vivarium on a 12 h light/dark cycle with their littermates. Flexible probe implantations were performed similar to silicon probe implantation with some variations.^[^ ^53^ ^]^ Animals were anesthetized with isoflurane. A craniotomy was performed under stereotaxic guidance at 2 mm posterior of bregma and 1.5 mm to the left of midline. A ground screw electrode was placed over cerebellum. Once the craniotomy was made it was temporarily covered with gel foam. The base of a custom‐made 3D‐printed head cap was cemented to the skull using dental cement.^[^ ^53^ ^]^ The craniotomy was then exposed, and a small needle was used to break the dura mater. A 20 µm glass virus injection pipette (Drummond, #3‐000‐203‐G/X) was secured to a custom‐made pipette stereotaxic holder. The flexible probe was attached to the virus injection pipette using 10 000 g mol^−1^ of Poly(Ethylene Glycol) (PEG) below and above the electrode contact sites (Figure S8, Supporting Information). The electrode was connected to an Intan RHD2000 interface board. Signals were digitized by the head‐mounted preamplifier that was grounded to the skull screw and electrode. Electrophysiological recording was conducted during implantation to guarantee proper placement of the electrode. The pipette and electrode were inserted into the brain at a rate of 250 µm min^−1^ until it reached a final depth of ≈800 µm. If there was unit activity present in more than 5/32 channels with spikes of 80 µV or greater, the electrode would be attached to the skull using super glue (Loctite, Super Glue Control Gel). Once the superglue cured completely, the virus injection pipette was removed (waited for at least 10 min – the PEG dissolving time). If there was no single unit activity, the electrode and pipette would be removed together and reinserted in the same craniotomy (200 µm posterior) until it was secured in layer V of the cortex and the unit criteria was met. The craniotomy was covered with sterile mineral wax and the remaining two walls of the 3D printed hat were cemented to the base thereby enclosing the implant.^[^ ^53^ ^]^ Parafilm was used to cover the top of the hat and to protect the electrode.

### Recording

After 3 days of postsurgery recovery animals were recorded for over 150 days at irregular intervals. Implanted animals were single housed animals and remained in the facility except on recording days where they were in a quiet brightly dimmed room and recorded in their home cage seated above a copper mesh grounded to the recording system. Long‐term electrophysiological recordings were conducted using an Intan RHD2000 interface board and 32‐channel digital head stages with sampling at 20 kHz (Intan Technologies, Los Angeles, CA). Recording sessions ran for 1–3 h where animals had unlimited access to food. Kilosort spike‐sorter was used to isolate units and sessions were manually curated to guarantee appropriate spikes sorting. The quality and quantity of units were analyzed over time. Histopathology analysis was not performed on these brains because we focussed on electrophysiological performance of the devices (detailed biostability and structural biocompatibility evaluation of the probes were performed earlier by Vomero et al., 2022,^[^ ^20^ ^]^).

### Single Unit Analysis

A concatenated signal file was prepared by merging all recordings from a single animal from a single day (unless otherwise noted for “same” neuron analysis). Putative single units were first sorted using Kilosort^[^ ^54^ ^]^ and then manually curated using Phy to guarantee appropriate spikes sorting (https://phy‐contrib.readthedocs.io/). Phy's software allows to manually curate the clusters and labeled them as noise or as single units. All single units are used in the analysis. The quality and quantity of units were analyzed over time using a custom MATLAB (Mathworks, Natick, MA) script.

### Cell‐Type Classification

In the processing pipeline, cells are classified into three putative cell types: narrow interneurons, wide interneurons, and pyramidal cells. Interneurons are selected by 2 separate criteria; narrow interneuron is assigned if the waveform trough‐to‐peak latency is less than 0.425 ms. Wide interneuron is assigned if the waveform trough‐to‐peak latency is more than 0.425 ms and the rise time of the autocorrelation histogram is more than 6 ms. The remaining cells are assigned as pyramidal cells.^[^ ^55^, ^56^, ^57^ ^]^ Autocorrelation histograms are fitted with a triple exponential equation to supplement the classical, waveform feature based single unit classification (https://cellexplorer.org/pipeline/cell‐type‐classification/).^[^ ^58^ ^]^ Bursts were defined as groups of spikes with interspike intervals < 9 ms. 22 putative single units per session from 2 animals in 49 sessions were isolated.

### Crosstalk Measurements

**Figure 6:**
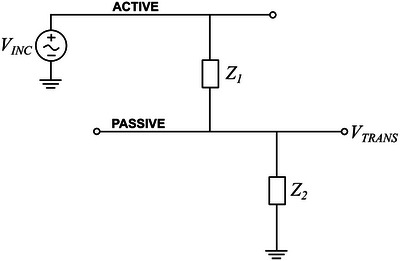
Model of the circuit used for the crosstalk measurements using a two‐port network analyzer. *V*_INC_ and *V*_TRANS_ correspond to the incident and transmitted signal, respectively, *Z*_1_ stands for the coupling impedance between interconnects and *Z*_2_ represents the high‐impedance input of the analyzer.

Network analyzers are instruments specially devised to characterize the electrical behavior of linear electrical networks, particularly two‐port networks, such as the case of a pair of interconnects running in parallel, i.e., a transmission line. As depicted in **Figure** **6**, the analyzer measures the incident waves entering the active line (VINC) and the transmitted waves exiting the passive line (VTRANS) and computes the transfer function based on the signal attenuation. The smaller the attenuation between incident and transmitted waves (in absolute value), the higher the coupling through Z1, i.e., the higher the crosstalk (−∞ dB corresponds to zero coupling and 0 dB corresponds to 100% coupling). Crosstalk was here quantified using a two‐port network analyzer (Agilent 4395A, Keysight Technologies, CA). A test set (Agilent 87512A, Keysight Technologies, CA) was used to connect source and receiver to the device under test (DUT) and a high‐impedance input adapter (Agilent 41802A, Keysight Technologies, CA) was plugged in the receiver (Z2). The source was connected to the left end of one track (active line) and the receiver to the right end of the second track (passive line). The transfer function was acquired from 100 Hz to 1 MHz at a bandwidth of 10 Hz for an input voltage of 100 mV. A Faraday cage was used to reduce electromagnetic interference.

### Statistical Analysis

Statistical analyses were performed with MATLAB functions or custom‐made scripts. No specific analysis was used to estimate minimal population sample or group size, but the number of sessions, and recorded cells per channel were similar to those employed in previous related works.^[^ ^18^ ^]^ The unit of analysis was either single neurons or sessions within animals, and this is stated in the text. Unless otherwise noted, nonparametric two‐tailed Wilcoxon rank‐sum (equivalent to Mann–Whitney U‐test) or Kolmogorov–Smirnov test was used. On box plots, the central mark indicates the mean, error bars indicate one standard deviation. The experimenter was not blind to the type of electrode. Unless otherwise stated, all electrochemical data is reported as mean±SD, related box‐plots show the interquartile range (IQR) as box, whiskers denote the area within 1.5 IQR, the central mark shows the mean and outliers are marked with diamonds.

## Conflict of Interest

The authors declare no conflict of interest.

## Author Contributions

C.B., M.V., and M.S. contributed equally to this work. G.B. proposed the design which was realized by C.B., M.V., and R.L. who developed the microfabrication protocol with the support of T.S. and M.A., M.S. and M.V. planned and performed the in vivo experiment, immunohistochemistry, and electrophysiological analysis with the support of G.B. M.V. and M.C. fabricated the ceramic interconnection and performed the cross‐talk analysis. C.B. performed electrode coatings, charge injection analysis, and designed and performed the explant analysis protocols with the support of M.A. M.A. compiled the manuscript to which all authors provided their scientific analysis and critical input.

## Supporting information

Supporting Information

## Data Availability

The data that support the findings of this study are available from the corresponding author upon reasonable request.
