# Supplementary material for: Multilayer Arrays for Neurotechnology Applications (MANTA): Chronically Stable Thin‐Film Intracortical Implants
Source: Adv Sci (Weinh). 2023 Mar 19;10(14):2207576. doi: 10.1002/advs.202207576 (PMC10190290; doi:10.1002/advs.202207576)
Supplement: Supplementary file 1 — Supporting Information [file ADVS-10-2207576-s001.pdf]

ADVANCED SCIENCE
Open Access

Supporting Information

for *Adv. Sci.*, DOI 10.1002/advs.202207576

Multilayer Arrays for Neurotechnology Applications (MANTA): Chronically Stable Thin-Film Intracortical Implants

*Christian Böhler, Maria Vomero, Marisol Soula, Mihály Vöröslakos, Maria Porto Cruz, Rickard Liljemalm, György Buzsaki, Thomas Stieglitz and Maria Asplund**

Supporting Information

Determination of performance metrics for high-density neural probes

We first separate the "recording section", of the probe, defined as the part of the probe where electrodes are placed, and the "cable section", defined as the part of the probe leading from the brain surface to the electrodes. The reason for this is that they represent separate aspects of integration density. For the recording section we calculated the percental coverage of electrodes (ratio between area covered by electrodes and area covered by insulation) and the total area and volume of the recording section normed by the total number of electrodes (area and volume per electrode). For probes where electrodes were distributed over more than one cluster, we defined several recording sections, so that the added insulation between the clusters would not negatively impact the estimated integration density. For the cable section we calculated the cross-section normed with the number of electrodes (cross-section area per electrode). The overall length of the cable is not important as this can be changed arbitrarily in the lithography process. The same is true for any additional part of the probe extending beyond the recording section e.g. tip shape.

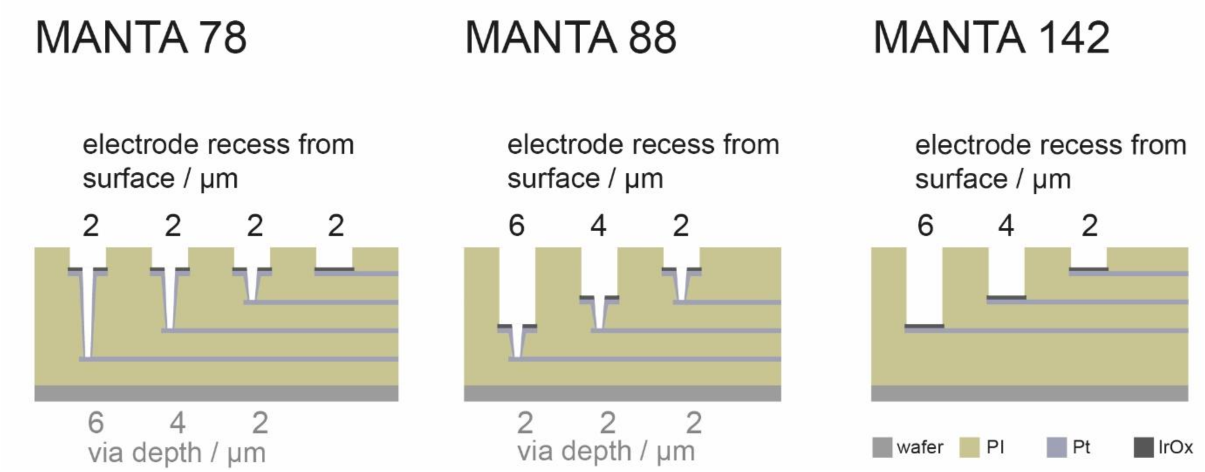

Supporting Figure S1. Layout variation of MANTA. Cross-sectional view through the different MANTA designs, illustrating the electrode recess from the probe surface, as well as the different via-depth for the individual probe styles. Dimensions are not to scale.

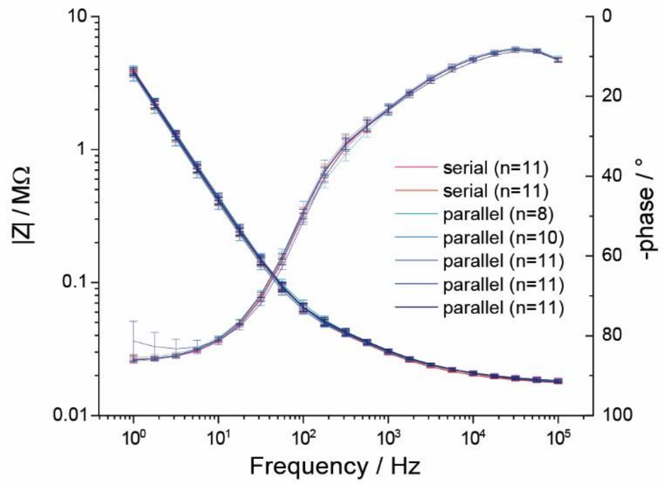

Supporting Figure S2. Parallel vs sequential PEDOT-deposition. EIS characteristics of PEDOT/PSS coated electrode sites when deposition is performed sequentially on each individual electrode site (serial), or when multiple electrode sites are shorted for parallel deposition. Variability in impedance (measured at 1 kHz) was found to be less than 2%.

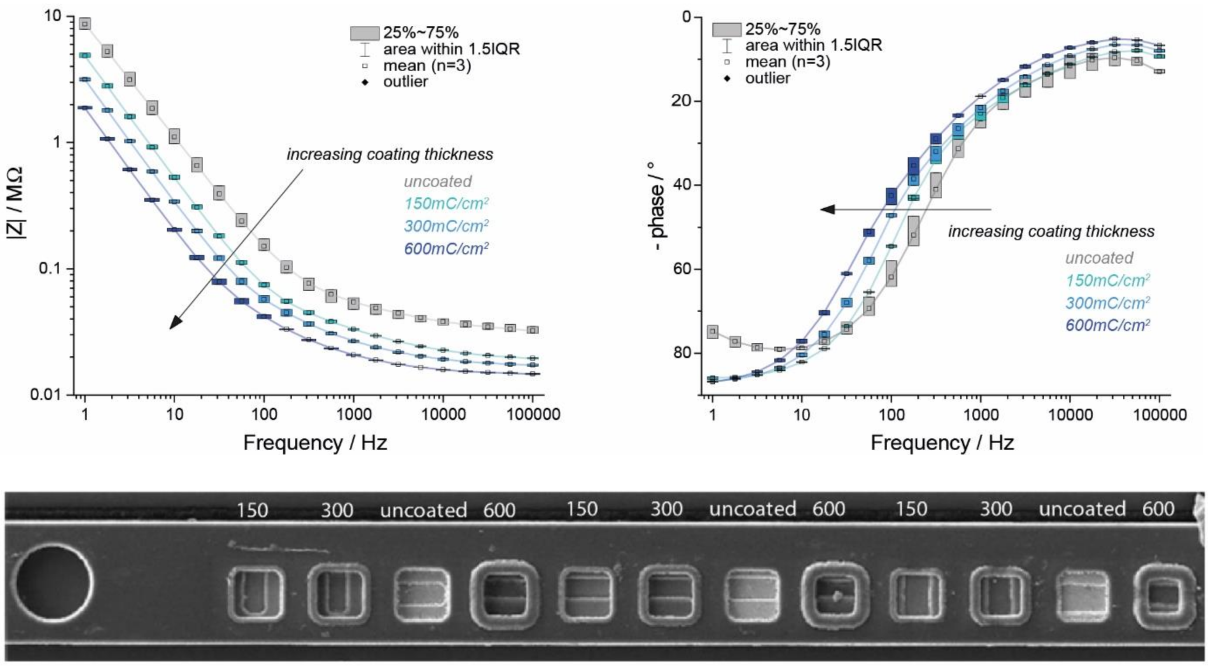

Supporting Figure S3. PEDOT/PSS at varied deposition charge. Upper graphic: EIS characteristics of PEDOT/PSS sites with different PEDOT thickness, here denoted in terms of polymerization charge. Lower graphic: SEM of coatings with different thickness. Numbers indicate total deposition charge in mC/cm^2.

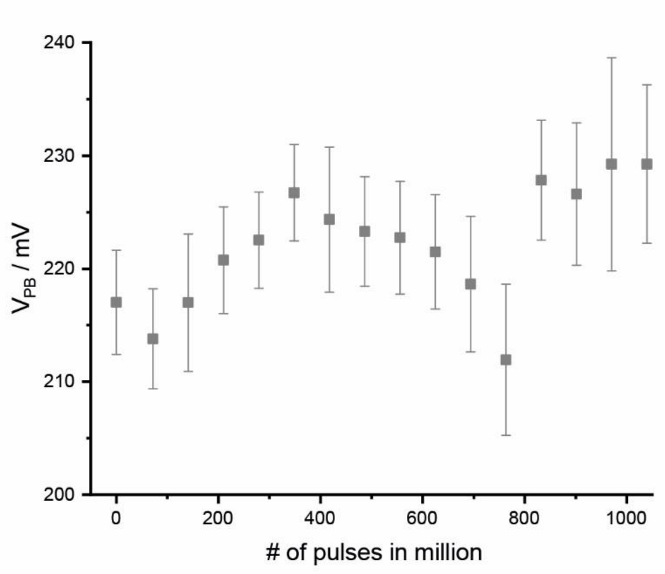

Supporting Figure S4. Electrode polarization during pulsing. Variation of the transient voltage recording (V_{PB}) during continuous biphasic pulsing at a charge density of 1mC/cm^2. Fluctuations (within 10%) are in line with temperature variations observed inside the laboratory during the pulsing time.

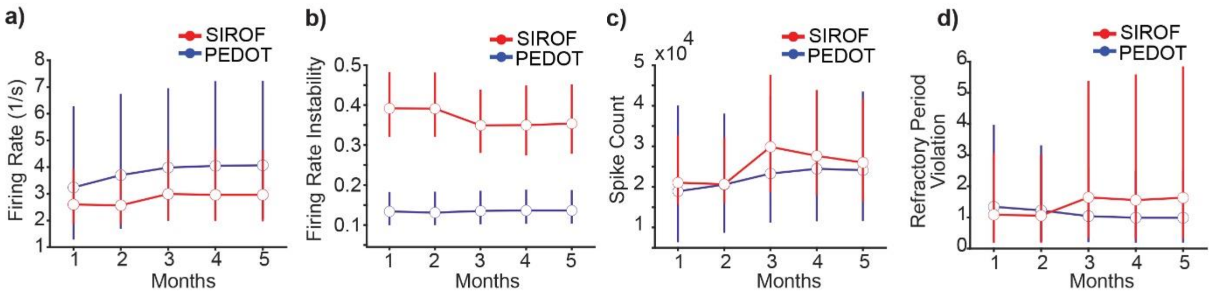

Supporting Figure S5. Electrode properties. Longitudinal analysis of firing rate parameters: firing rate (**a**), firing rate instability: the mean of the absolute differential "firing rate across time" divided by the mean abs(diff(firingRateAcrossTime)). (**b**), spike count (**c**) and refractory period violation: Fraction of Inter-spike intervals less than 2 ms. (**d**) did not change significantly over 5 months (n = 15 and 34 sessions for SIROF and PEDOT electrodes, respectively, $p > 0.05$, Kolmogorov-Smirnov test) in PEDOT and SIROF electrodes.

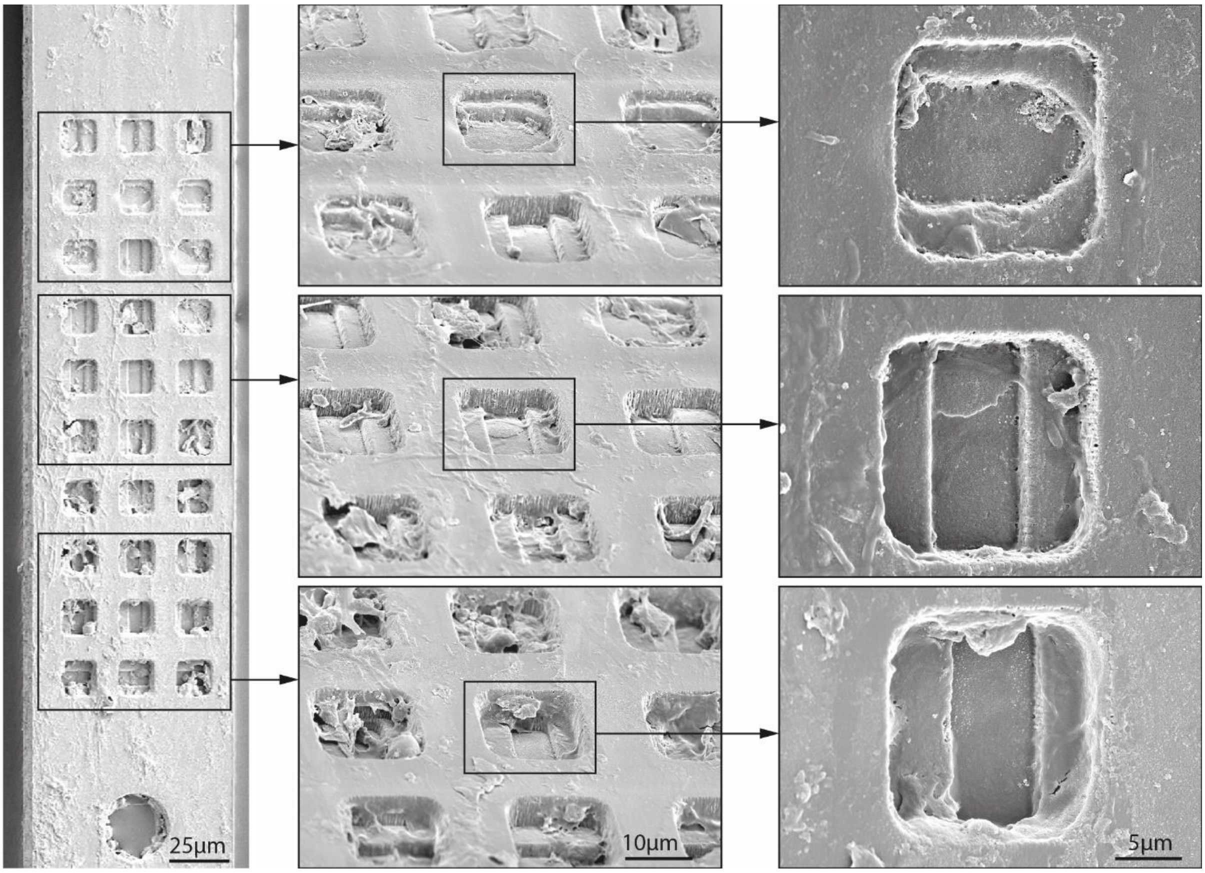

Supporting Figure S6: Explant analysis. Representative SEM images of the explanted IrOx probe showing intact electrode sites with some biological residues. Different recess depths for the electrode sites in relation to the individual metallization planes can be identified in the three panels in the middle (the most superficial via shown at the top, the deepest via showing in the bottom picture).

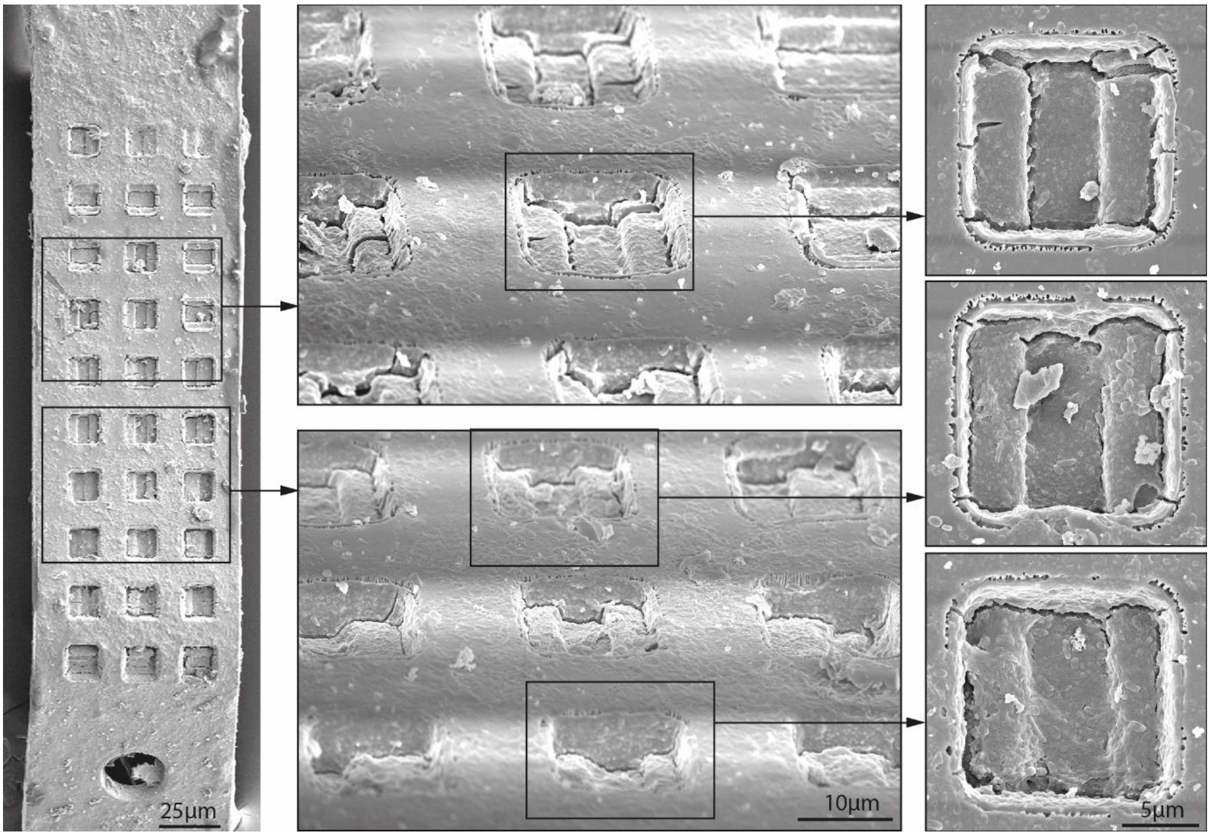

Supporting Figure S7: Explant analysis. Representative SEM images of the explanted PEDOT probe showing electrode sites with minor crack formation in the PEDOT coating, likely in relation to the SEM preparation. Probes do not show any signs of delamination of the polyimide-polyimide interface in between the layers and full probe integrity is maintained.

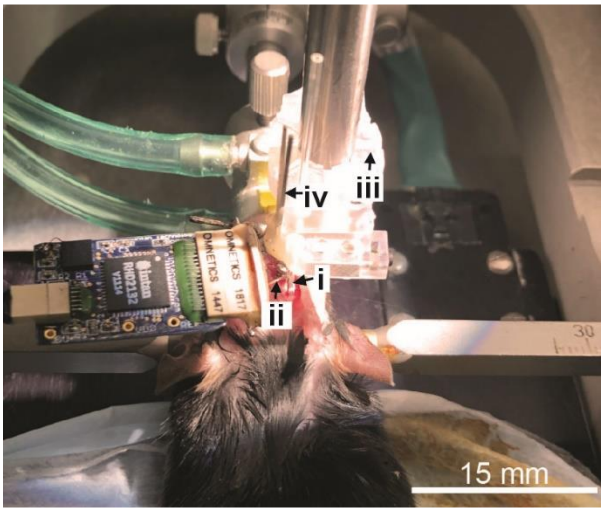

Supporting Figure S8. Set up. a) Intraoperative photograph of a mouse during implantation of a probe with simultanous recording of neural activity. (i= flexible probe; ii= Glass pipette used to insert the flexible probe; iii= 3D printed holder for probe and pre-amplifier. iv= metal bar used to attach pre-amplifier during insertion).
